# A method for the unbiased and efficient segmental labelling of RNA-binding proteins for structure and biophysics

**DOI:** 10.1038/s41598-017-13950-8

**Published:** 2017-10-26

**Authors:** Christopher Gallagher, Fabienne Burlina, John Offer, Andres Ramos

**Affiliations:** 1 Institute of Structural and Molecular Biology, University College London, London, WC1E 6XA UK; 2 The Francis Crick Institute, London, NW1 1AT UK; 3 Sorbonne Universités, UPMC Univ. Paris 06, École Normale Supérieure, PSL Research University, CNRS, Laboratoire des Biomolécules (LBM), 4 place Jussieu, Paris, 75005 France

## Abstract

Most eukaryotic RNA regulators recognise their RNA and protein partners by the combinatorial use of several RNA binding domains. Inter-domain dynamics and interactions play a key role in recognition and can be analysed by techniques such as NMR or FRET, provided that the information relative to the individual interactions can be de-convoluted. Segmentally labelling the proteins by ligating labelled and unlabelled peptide chains allows one to filter out unwanted information and observe the labelled moieties only. Several strategies have been implemented to ligate two protein fragments, but multiple ligations, which are necessary to segmentally label proteins of more than two domains, are more challenging and often dependent on the structure and solubility of the domains. Here we report a method to ligate multiple protein segments that allows the fast, high yield labelling of both internal and end domains, depending on the requirements. We use TCEP and mercaptophenylacetic acid (MPAA) in an optimised reaction environment to achieve an efficient ligation of protein domains independently from their structure or solubility. We expect the method will provide a useful tool for the molecular study of combinatorial protein–RNA recognition in RNA regulation.

## Introduction

A molecular insight into the interaction between multi-domain RNA binding proteins and their targets is essential to understand RNA regulation of gene expression. The majority of eukaryotic RNA binding proteins interact with their RNA and protein partners using multiple RNA binding domains. These domains cooperate to select the proteins’ RNA targets and regulate their metabolism. Multiple domains can either interact with RNA as a preformed rigid unit, make contact upon RNA binding, or recognise different RNA sequences in a combinatorial fashion^1,2^. Inter-domain dynamics play a key role in these functional interactions: they allow adaptation to differently structured targets, mediate fly-casting or conformational selection mechanisms of binding, and can be used to establish an equilibrium between competing pathways^3^. Importantly, both the RNA-binding domains and the inter-domain linkers have essential roles in these dynamics and contribute to the overall interaction^4^.

FRET, NMR and Small Angle Neutron Scattering (SANS) experiments provide entry points to describe the structure and dynamics of protein-RNA regulatory complexes^3^. However, labelling of individual amino acids or larger protein segments is often necessary to define the molecular basis of the protein-RNA interactions^5^. In NMR and SANS, ligating stable-isotope (e.g ^15^N, ^13^C, ^2^H) labelled protein fragments to unlabelled fragments allows the user to focus the experiment on the (labelled) fragment under investigation within the larger structure, thus filtering out unwanted observables. In addition, ligating modified peptides within large proteins allows insertion of unnatural amino acids and chemical reporters into multi-domain systems which is useful, for example, in FRET experiments. Interestingly, ligation also allows the joining of protein and nucleic acid chains^6^. This may be used to stabilise weak complexes and facilitate their structural study.

Segmental protein labelling first became possible with the development of native chemical ligation (NCL)^7^. NCL couples two peptide chains together, one containing a *C*-terminal thioester, the other an *N*-terminal cysteine residue. Expressed protein ligation (EPL)^8^ is a variant of NCL where the peptide thioester is obtained by biological recombinant expression of the protein by using naturally occurring inteins to generate the thioester. A further development of EPL is trans-splicing^9,10^ which harnesses the phenomenon of split proteins, where two complementary split-intein fragments are fused to the sequences to be ligated. Mixing samples of the two fusion proteins results in the two intein fragments forming one single structural unit that is able to autocatalyze its removal while ligating the two flanking protein fragments in the process. The power of EPL can be enhanced by combining this method with, for example, enzymatic synthesis, resulting in a complete labelling procedure^11^. More recently, other enzymes have been used to join two protein fragments. These include Butelase^12^, and the evolutionary related asparaginyl endopeptidase OaAEP1, which can be engineered to achieve high ligation efficiency^13^. They also include the bacterial enzyme Sortase, which can been used to join two protein fragments carrying the Sortase recognition motif^14,15^. However, these ligation strategies have different strengths and limitations and the choice of method is dependent on the system to be investigated. For example, RNA-binding domains are generally too large to be chemically synthesized and indeed NCL is normally performed on relatively short peptides^16^. Sortase-mediated ligation inserts a tag of up to nine non-native amino acids in the wild type sequence that can potentially alter the properties of the native domains or the inter-domain linkers and interfere with RNA target recognition^5^.

Many RNA-binding proteins are comprised of more than two RNA-binding domains and the separate labelling of both end and internal domains of the protein is often required to answer the relevant biological questions. The labelling of an internal domain of a multi-domain protein requires at least two ligation events. The general value of establishing protocols that allow the ligation of multiple domains in the segmental labelling of multi-domain proteins has been readily recognised in two early studies that provide a proof-of-principle for the insertion of labelled protein segments in a multi-domain protein by either trans-splicing^17^ or, EPL^18^. However, high efficiency trans-splicing also requires optimisation of the sequences close to the splicing junction^5,19,20^, while the yield of EPL is system dependent and can vary in the different steps of a multi-step ligation. This has limited the application of EPL, and later protein ligation studies have mainly focused on single step ligations.

Here we describe a method to label selectively either end or internal protein segments of RNA binding proteins. The method is efficient and is independent of the protein structure or inter-domain contacts. Our strategy is based on applying our recent advances in chemical ligation of small synthetic peptides^21^ to the ligation of larger recombinant protein chains. Chemical ligation is typically performed under denaturing conditions to decouple it from structure-dependent variations and to provide a consistent high solubility for the fragments to be ligated. Importantly, the ease of refolding of the most common RNA binding domains (e.g. K-homology (KH), RNA Recognition Motif (RRM), Zinc Finger (ZnF) domains)^22–25^ as well as protein constructs containing two or more such domains, makes refolding of the intermediate and final product viable. We have tested the method on a ~30 kDa three-domain construct comprising the three amino terminal K-homology domains (KH) of the RNA-binding protein: KH-type splicing regulatory protein (KSRP)^26^. We show that a combination of 4-mercaptophenylacetic acid (MPAA) as a thiol additive, and tris(2-carboxyethyl)phosphine hydrochloride (TCEP) as a reducing agent allows a fast high yield reaction with protein concentration in the sub-millimolar range. These conditions are similar to that of ligations involving short peptides and therefore suggest that this protocol could be used in the segmental labelling of a broad range of RNA binding proteins.

## Results

**Figure 1:**
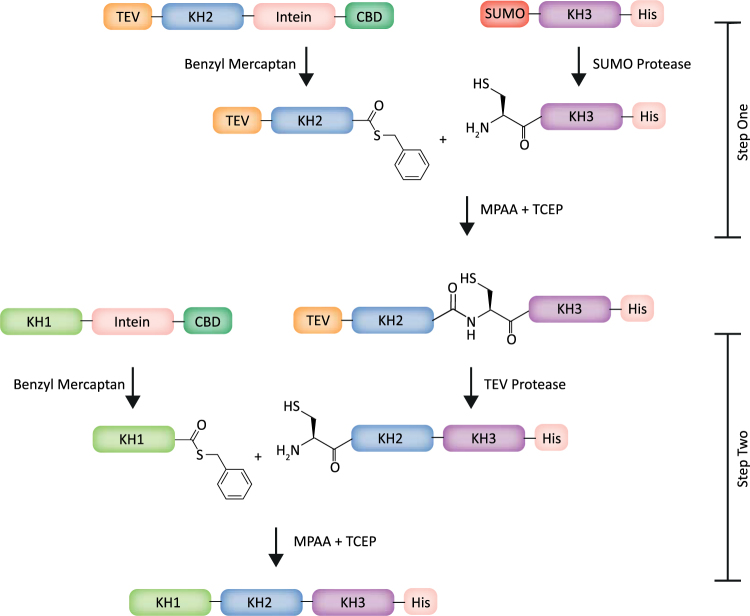
Workflow of the expressed protein ligation protocol for the KH1, KH2, and KH3 RNA binding domains of the RNA regulator protein KSRP. The domains are expressed as either intein-CBD or SUMO-His fusion proteins and purified by chitin and nickel agarose affinity resins respectively. Intein fusion proteins are released from the chitin affinity resins by the strongly nucleophilic thiol additive benzyl mercaptan, which results in benzyl thioester formation at the *C*-terminus of the released protein. Instead, proteolytic cleavage catalysed by either SUMO or TEV proteases form *N*-terminal cysteine species. The ligations are performed at 40 °C in 6 M guanidine, pH 6.5 using TCEP as reducing agent and MPAA as a catalyst.

We report a detailed protocol for the efficient segmental labelling of RNA binding proteins. The method combines technologies from chemical peptide ligation and EPL to sequentially ligate three protein domains into a single chain (Fig. 1). Ligations were performed in denaturing buffer in order to eliminate any variations in the reaction due to the structure of the system(s) and to reach high concentrations for efficient reaction. Ligations were monitored to completion by analytical HPLC. Real time monitoring was useful to minimize unwanted side reactions such as cysteine oxidation and hydrolysis of the thioester. The key elements to obtaining efficient ligation that is both kinetically fast, and high yielding, were the use of MPAA as a thiol exchange catalyst and TCEP as a reducing agent as well as the optimisation of the reaction parameters using analytical HPLC monitoring.

### Cloning and expression of the protein domains

**Figure 2:**
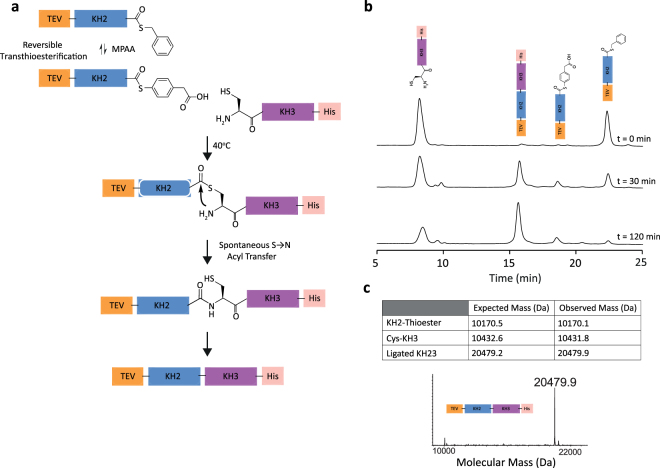
KH2-KH3 ligation. (**a**) Schematic of the ligation reaction representing the highly reactive KH2 MPAA thioester reacting with the *N*-terminal cysteine of KH3 in 6 M guanidine, pH 6.5 and in the presence of MPAA and TCEP at 40 °C. (**b**) HPLC traces showing the formation of the MPAA thioester intermediate and ligated KH23 product (identified by cartoons) over time. Experiments were repeated three times. (**c**) (Upper) Expected and measured masses of the reagents and product of the ligation. For the Cys-KH3 a 98% ^15^N labelling has been assumed based on the level of enrichment of the ^15^N nitrogen source. (Lower) Reconstituted electrospray mass spectrum showing the de-convoluted molecular mass of the ligated KH23.

KSRP is a multi-domain multi-functional protein that regulates both the stability of mRNAs containing AU-rich elements in their 3′ untranslated regions, and the biogenesis of selected miRNAs^27,28^. In the protein ligation protocol described here we join three KH domains of KSRP (KH1, KH2, and KH3) to form a single protein chain. The three domains were expressed as individual fusion proteins (Figure S1a,b and c). KH1 and KH2 were flanked by an intein-chitin binding domain (CBD) to facilitate their purification and for KH3, a Histag was added for the same purpose. The residues located at the junction site in the fragments to be ligated were mutated: in both KH1 and KH2 derivatives, the amino acid immediately preceding the thioester function was replaced by a glycine to improve ligation kinetics, and a cysteine was introduced on the *N*-terminus of both KH2 and KH3 derivatives to allow NCL^29^. TEV and SUMO cleavage recognition sites were included in KH2 and KH3 respectively to allow protection and unmasking of the *N*-terminal cysteines when required. A SUMO domain was used to exemplify that different types of fusion proteins can be used for this method. First, KH3 was cloned in a modified pNIC vector where the domain is sandwiched between a *C-*terminal Histag and an *N*-terminal SUMO tag. The protein was purified using a nickel agarose matrix and the SUMO tag cleaved by SUMO protease, exposing the *N*-terminal cysteine (Figure S1c) that reacts with the KH2 thioester (Figs 1 and 2). KH2 was cloned in a pTWIN1 intein-CBD vector, sandwiched between the *C*-terminal intein-CBD, whose cleavage by thiolysis gives the thioester derivative used in the first ligation step, and an *N*-terminal non-canonical TEV cleavage site (QNLYFQ/C) masking the *N*-terminal cysteine. The expressed KH2 protein was initially purified using a chitin agarose column that binds the CBD domain in a non-reversible (at neutral pH) manner. With the KH2 protein attached to the chitin column, buffered benzyl mercaptan was added to cleave KH2 off column by thiolysis (Figure S1b). Benzyl mercaptan is a strong nucleophile which favours the thioester exchange of the KH2 from the intein. It is also a poorly activated thioester and is therefore fairly stable to hydrolysis^30^. This was an important consideration because of the relatively long period required to achieve efficient cleavage from the chitin column. The thioester function was later used to ligate KH2 to the *N*-terminal cysteine of a KH3 partner during the first step of the ligation procedure (Fig. 1), as described below. KH1 was cloned into a pTWIN1 vector and purified on a chitin column as for KH2. In the experiment described here, KH1 and KH2 were expressed in LB media overnight to obtain un-labelled proteins, while KH3 was expressed in M9 minimal media with ^15^N NH_4_CL as the only nitrogen source to obtain a ^15^N labelled protein, to be used as reporter in NMR analysis. The proteins were expressed at high level and expression and purification procedure is detailed in the Materials and Methods section.

### Purification of the protein domains and ligation of KH2 and KH3

Cleavage of the intein from KH2 was induced by the addition of benzyl mercaptan as previously described^30^ (Fig. 2a). We found that optimal cleavage conditions were obtained with thoroughly degassed buffer held at pH 7.0. The pH is kept low to suppress hydrolysis of the thioester formed upon intein cleavage, even though the rate of thiolysis is also lower at this pH. After 16 hours the KH2 thioester was purified from the cleavage reaction by semi-preparative reverse phase HPLC on a C18 column and characterized by MALDI-TOF MS. Peak fractions were flash-frozen to minimize hydrolysis and the presence of the thioester group was confirmed by electrospray mass spectrometry (Figure S1b).

The SUMO-KH3-Histag construct was eluted from the nickel matrix using imidazole and the KH3-Histag fragment cleaved from the SUMO tag using SUMO protease following the manufacturer protocol. Use of a tag to protect the *N*-terminal cysteine rather than expressing the domain in a commercial intein vector has been reported to prevent untimely cleavage and unmasking of the reactive cysteine in bacteria: reviewed by Michel and Allain^5^. We found SUMO cleavage of our KH3 construct to be specific and efficient. This is important as it minimises the time required for cysteine deprotection and therefore the time the free *N*-terminal cysteine spends in aqueous solution. Indeed, the *N*-terminal cysteine is readily capped if traces of aldehydes or ketones are present, preventing ligation. The unmasked KH3 was purified from the SUMO cleavage reaction using semi-preparative HPLC reverse phase purification and flash-frozen. The free Cys-KH3 was checked using electrospray mass spectrometry (Figs 2c and S1c).

KH2 and KH3 were dissolved in 6 M guanidine ligation buffer to a ~0.5 mM concentration and combined to initiate the ligation reaction. The small difference in KH2 and KH3 concentration in our HPLC chromatogram (Fig. 2b) are due to inaccuracies in handling the small amount of lyophilised protein. Aliquots were collected at time points throughout the reaction, which was close to completion at 120 minutes (Fig. 2b). This is an order of magnitude faster than previously reported for an equivalent ligation of similarly sized domains^25^. The speed of the reaction is in fact similar to what we observed for short peptides in the presence of MPAA additive and TCEP^31^. The use of HPLC allowed an accurate monitoring of the reaction, including the concentration of the MPAA-activated thioester, which is the most reactive thioester species in the mixture. The reaction was terminated with hydroxylamine hydrochloride which reacts with any remaining thioester groups which could otherwise form branched products. Ligated KH23 protein was refolded as described in the Materials and Methods.

### KH1 and KH23 ligation

**Figure 3:**
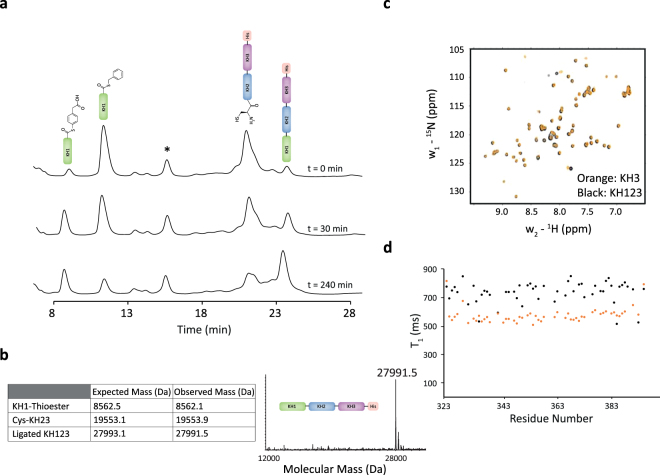
KH1-KH23 ligation. (**a**) HPLC traces showing the formation of the MPAA thioester intermediate and ligated KH123 product (identified by cartoons) over time. Diphenyl column was used to resolve the KH23 and KH123 peaks. The unidentified ‘*’ impurity, which is at constant concentration during the reaction, provides a serendipitous control. Experiments were repeated three times. (**b**) (Left) Expected and measured masses of the reagents and product of the ligation. For the KH3 domain a 98% ^15^N labelling has been assumed based on the level of enrichment of the ^15^N nitrogen source. (Right) Reconstituted electrospray mass spectrum showing the de-convoluted molecular mass of the ligated KH123. (**c**) Superimposition of the backbone amide of ^1^H{^15^N} correlation experiments recorded on the KH3 and ligated KH3-only ^15^N labelled KH123 proteins, indicating that the KH3 domain is correctly folded and assembled in the KH123 construct and highlighting the high quality of the NMR data. (**d**) The values of the ^15^N longitudinal relaxation time of backbone amide groups in the ligated KH3 (black dots, this study) and of the isolated domains (orange dots, as previously reported^38^) plotted along the protein sequence.

The cleavage of the intein tag of KH1 to form a *C*-terminal thioester was obtained by addition of benzyl mercaptan (Figs 1 and S1a) using the same protocol described above for KH2^30^. After cleavage the KH1 thioester was HPLC purified and freeze dried. The purified KH23 was efficiently refolded by step dialysis from guanidine ligation buffer to a TEV cleavage buffer. Correct refolding of the ligated KH23 fragment was confirmed using ^15^N-correlation 2D NMR experiments (data not shown). Proteolytic cleavage of the modified TEV site within the refolded KH23 construct was used to unmask the KH2 *N*-terminal cysteine residue for the second step ligation. As for the SUMO cleavage it is important that the TEV protease digestion is efficient. The Cys-KH23 was HPLC purified and freeze dried to be stored at −80 °C in a stable form. The ligation between KH1 and KH23 was performed using the same strategy and conditions used in the KH2 KH3 ligation; re-suspending KH1 and KH23 in denaturing ligation buffer and then combining the two proteins. Monitoring of the ligation showed that, the reaction was very efficient, with some of the product already detectable at the first time point and the reaction being almost complete after 4 h (Fig. 3a). This corresponds to only a ~2-fold difference in kinetics with the two-domain ligation. This difference can be explained by the larger size of the *C*-terminal fragment in the KH123 ligation. As an important technical note, the HPLC monitoring of the KH1 KH23 ligation reported here required a diphenyl column. The standard C18 column does not resolve the KH23 and KH123 peaks due to their relatively large molecular weights and similar amino acid compositions. The diphenyl column resolved the peaks of the ~20 kDa and ~30 kDa proteins, although the KH123 peak remains broad (Fig. 3a) and this makes it more difficult to precisely quantify the product, although most of KH23 has converted to the 3-domain product. After reaction termination the KH123 protein was refolded as described for KH23 and its structure confirmed using ^15^N-correlation 2D NMR spectroscopy (Fig. 3c). Finally, as a proof of principle for the quality of the data obtainable from this ligated, single domain-labelled protein, we recorded an NMR relaxation experiment and showed that accurate ^15^N T1 relaxation data can be obtained for 52 backbone amide groups in the ligated KH3 domain (Fig. 3d and Supplementary Table 1). This compares well with the 53 ^15^N T1 values obtained for the same groups from spectra of the isolated KH3 domain – with 50 of the resonances of the two data sets being in common (Fig. 3d), highlighting the completeness of the data obtainable from the reduced complexity spectra.

## Discussion

This manuscript describes a protein ligation method for the segmental labelling of multi-domain RNA-binding proteins. The method addresses a long-standing problem of heterogeneity and slow ligation kinetics that has hindered the labelling of the internal domains of multi-domain proteins and in turn, the structural and biophysical study of their dynamics and target recognition.

The ligation strategy we present here applies recent advances in NCL protocols^21,32^ to large multi-domain RNA-binding proteins. The use of catalytic thiol additives and reducing agents have significantly improved the efficiency of the ligation of short chemically synthesized peptides. Here we use them to ligate protein domains in denaturing conditions to establish an efficient ligation protocol that is not dependent on protein structure and can be used in multi-step ligations for the labelling of internal segments of multi-domain RNA binding proteins.

Our ligation reaction relies on MPAA as a catalyst additive and TCEP as the reducing agent for high speed kinetics. The addition of thiophenol to the ligation significantly increases ligation rate as the reactive peptide thiophenol ester is formed by thioester exchange, however, its effect is limited by solubility^33^. In contrast, MPAA is water soluble and can be added at high concentration, thereby increasing the concentration of activated MPAA thioester and accelerating ligation^32^.

Conditions reported here were optimised on the basis of careful monitoring of the reaction products and intermediates during the ligation using a combination of analytical HPLC and mass spectrometry (Figs 2c and 3b). We found that, in these conditions, the time required for ligation is tenfold less than what is reported in EPL ligation studies involving protein domains of similar size, in either folded^34^  or unfolded conditions^25^. Indeed, the reaction kinetics are comparable to those observed for the ligation of small peptides^29,31,32^. This has important practical implications, as side reactions leading to hydrolysed, inactive species are less likely if completion is achieved in a shorter time, and a faster reaction normally results in higher yield of ligated product. On a more general note, our results indicate that the unfavourable entropic effect of chain length on the speed of ligation does not prevent efficient inter-domain ligation in RNA-binding proteins of at least 30 kDa. One potential limitation of a ligation in unfolded conditions is that the domains need to be refolded after ligation and prior to structural and biophysical assay. However, as discussed above, the most common RNA binding domains are of small size and, generally, re-fold readily and independently. This is also true for most protein constructs comprising of a small number (2–4) of such domains that typically represent the RNA-binding regions in the proteins that regulate RNA metabolism. In ZnF and similar RNA-binding domains that chelate a metal ion, the ion can be added during re-folding. Importantly, the use of NCL is not traceless and the addition of cysteine to the *N*-terminus is an absolute requirement for the reaction. In this instance we also mutated the C-terminal thioester to glycine as this prevents the risk of epimerisation and normally increases the speed of reaction^29^. The cysteine mutation is normally tolerated and we show that it does not change the structure or the dynamics of the domain (Fig. 3c and d).

It is worth highlighting that, in contrast to published EPL studies of protein domains of similar size, the protocol described here does not require significant excess of one of the proteins, which testifies on its efficiency and simplifies the experimental strategy. Further a consistently highly efficient ligation protocol is an important factor in multi-step ligations. Finally, protein ligation in unfolded conditions should in principle be independent from the structure or sequence of the domain, with the only exception being the amino acids at the ligation junction. Indeed the similar kinetics observed for the two ligation reactions suggest the ligation protocol described here is not protein dependent.

To conclude, we present an efficient method for the segmental ligation of both end and internal domains of multi-domain RNA binding proteins. We expect this method will be used by the community in structural and biophysical studies to understand the molecular basis of RNA recognition by protein regulators. It is also worth mentioning that, although the method has been designed with RNA binding proteins in mind, it is applicable to other multi-domain proteins that refold efficiently.

## Methods

### Construct design and cloning

KH1 (residues T143-F223) and KH2 (residues H224-Q308) domains of human KSRP were cloned into a pTWIN 1 (New England BioLabs) vector using a standard PCR amplification and restriction enzyme digestion protocol to create the corresponding KH-intein-chitin constructs. For KH2 a modified TEV cleavage site (QNLYFQ/C) was also incorporated *N*-terminal to the domain, mutating H224 of KH2 into a cysteine. Finally, in both KH1 and KH2 the amino acid immediately preceding the intein fragment (F223 and Q308 respectively) was mutated to glycine to improve reaction kinetics using a QuickChange II Site-Directed Mutagenesis Kit (Agilent Technologies).

Human KSRP KH3 (residues G309-G396) was cloned into a modified pNIC (in house) vector containing an *N*-terminal SUMO tag using a ligase independent cloning (LIC) protocol. Primers were designed to incorporate a cysteine amino acid immediately after the SUMO recognition sequence resulting in G309 being mutated to a cysteine residue.

### Protein expression

Recombinant KH1 and KH2 fusion proteins were expressed in *E. coli* BL21(DE3) strain (Novagen, cat. no. 69450) growing in 100 µg/ml ampicillin LB media. Recombinant KH3 fusion protein was expressed in *E. coli* BL21(DE3) growing in 30 µg/ml kanamycin M9 minimal media and using ^15^N NH_4_Cl as the only nitrogen source.

4 L of growth media for each construct were inoculated to reach an OD_600_ of 0.1. The 4 L expression cultures were then incubated at 37 °C until an OD_600_ of 0.4 was reached. The temperature was then decreased to 22 °C and when an OD_600_ of 0.6 was reached protein expression was induced using IPTG at a final concentration of 0.5 mM (Generon). Protein expression continued overnight at 22 °C. Cells were harvested by centrifugation at 10,000 g for 10 minutes at 4 °C. Supernatant was removed and cell pellets stored at −80 °C.

### KH1-intein and KH2-intein expression and purification

Cells from 2 L of bacterial culture were re-suspended in 50 ml of ice cold lysis buffer containing 10 mM TRIS pH 8.0, 250 mM NaCl, 1 mM EDTA, 0.5 mM TCEP hydrochloride (Sigma, cat. no. 646547), Complete Ultra protease inhibitor tablet (Roche), DNase, and 1 mg/ml lysozyme. The suspension was sonicated on ice and centrifuged at 19,000 g for 50 minutes at 4 °C.

KH1 and KH2 fusion proteins were purified on a gravity flow chitin resin (New England BioLabs, cat. no. S6651L) column equilibrated with 10 bed volumes of chitin column wash buffer containing 10 mM TRIS pH 8.0, 250 mM NaCl, 1 mM EDTA, 0.5 mM TCEP. After loading the clarified cell lysate the column was washed with a further 2 × 10 bed volumes of chitin column wash buffer.

The KH domain was cleaved using 16% (v/v) benzyl mercaptan (Sigma, cat. no. B25401) in a buffer containing 10 mM TRIS pH 7.0, 250 mM NaCl, 1 mM EDTA. The buffer was prepared in a chemical fume hood as described by Welker. *et al*.^30^. The pH of the buffered solution was titrated to 7.0, the solution degassed, flushed with argon and a TCEP solution, pH 7.0 (Sigma, cat. no. 646547) was added to a final concentration of 16 mM. Two bed volumes of freshly degassed cleavage buffer was added to the protein-bound chitin matrix and incubated overnight at room temperature. Intein cleavage and thioester hydrolysis were assessed using mass spectrometry (Figure S1a, b).

After cleavage the elution fraction containing the activated KH1 or KH2 was concentrated using a viva spin column (Sartorius Stedim). The samples were then loaded on a semi-preparative reverse-phase Vydac C18 column (Grace) previously equilibrated with HPLC grade H_2_O, 0.1% trifluoroacetic acid (TFA), and 30% acetonitrile (ACN). Proteins were purified on a 30–60% ACN gradient over 30 min with a 5 ml/min flow rate. 2.5 ml fractions were collected and analysed by MALDI-TOF MS (Bruker, microflex series). The KH1 or KH2 fractions were flash frozen and lyophilised. Freeze dried samples were then stored at −80 °C for up to one month until required.

### SUMO-KH3 expression and purification

2 L bacterial pellets were re-suspended in 50 ml of ice cold buffer containing 10 mM TRIS pH 8.0, 200 mM NaCl, 10 mM Imidazole, 0.5 mM TCEP, Complete Ultra protease inhibitor, DNase, 1 mg/ml lysozyme, and lysed via sonication and clarified as above.

Clarified supernatant was loaded onto a Ni-NTA agarose resin column (Qiagen, cat. no. 30230) pre-equilibrated with: 10 mM TRIS pH 8.0, 1 M NaCl, 10 mM imidazole, 0.5 mM TCEP. Protein bound to the Ni-NTA agarose resin was washed with 10 bed volumes of wash buffer containing 10 mM TRIS pH 8.0, 1 M NaCl, 10 mM imidazole, 0.5 mM TCEP, followed by a further 10 bed volumes of wash buffer two containing 10 mM TRIS pH 8.0, 1 M NaCl, 30 mM imidazole, 0.5 mM TCEP. Washed protein was then eluted in 5 bed volumes of elution buffer containing 10 mM TRIS pH 8.0, 1 M NaCl, 300 mM imidazole, 0.5 mM TCEP.

Eluted fractions were dialysed against a 1:100 ratio of sample volume to dialysis buffer containing 20 mM TRIS pH 8.0, 150 mM NaCl, 0.5 mM TCEP overnight at 4 °C using dialysis tubing (SpectrumLabs). The dialysed sample was then concentrated using a viva spin column (Sartorius Stedim). Addition of SUMO protease (Invitrogen, cat. no. 12588) and incubation at 30 °C in 20 mM TRIS pH 8.0, 150 mM NaCl, 0.5 mM TCEP, 0.2% NP-40 (Sigma, cat. no. 74385) removed the SUMO tag.

Once digestion was >80% complete the sample was HPLC purified on a pre-equilibrated semipreparative C18 reverse phase column with HPLC grade H_2_O, 0.1% TFA, and 25% ACN and purified using a 25%–50% ACN gradient over 30 min with a 5 ml/min flow rate. Fractions were again analysed by MALDI-TOF MS. KH3 fractions were flash frozen and lyophilised. Freeze dried samples were then kept at −80 °C for up to one month until required.

### KH2 and KH3 ligation

Ligation reactions were carried out at an approximate KH1:KH2 ratio of 1:1. The ligation buffer was prepared from a 6 M guanidine hydrochloride, 200 mM sodium phosphate pH 6.5 and 2 mM EDTA solution. The solution was degassed under vacuum and flushed with argon. TCEP hydrochloride (Sigma, cat. no. C4706) was dissolved into the buffer under argon to a final concentration of 30 mM and the pH adjusted to 6.0. The buffer was degassed before adding 4-mercaptophenylacetic acid (MPAA)^32^ (Sigma, cat. no. 653152) to a final concentration of 60 mM. Finally the pH was adjusted to a value of 6.5.

KH2 and KH3 were dissolved separately into a volume of ligation buffer equal to half of the total reaction volume to obtain a final concentration of 0.5 mM (minimum). The two proteins were then combined and incubated at 40 °C until the ligation reached completion. Monitoring of the reaction was carried out by adding 1 µl of the reaction at different time points to 9 µl of 0.1% TFA in H_2_O. Time points were then loaded onto a Vydac C18 reverse-phase analytical column pre equilibrated with HPLC grade H_2_O, 0.1% TFA, and 35% ACN. A gradient of 35–50% ACN over 30 min at 1 ml/min flow rate was used to separate reactive species from ligated product.

After completion, hydroxylamine hydrochloride (Santa Cruz, cat. no. sc-211616A) pH 6.0 was added to the ligation reaction to a final concentration of 10 mM to hydrolyse any remaining thioester groups.

### Refolding and TEV cleavage of ligated KH23

The KH23 construct was readily refolded via a three-step dialysis. The sample was first dialysed into 3 M guanidine hydrochloride, 10 mM TRIS pH 8.0, 250 mM NaCl, 0.5 mM TCEP, 0.5 mM EDTA. Before being placed in 1 M guanidine hydrochloride, 10 mM TRIS pH 8.0, 150 mM NaCl, 0.5 mM TCEP, 0.5 mM EDTA. Finally the sample was dialysed into TEV digestion buffer containing 10 mM TRIS pH 8.0, 100 mM NaCl, 1 mM TCEP, 0.5 mM EDTA.

KH23 re-folding was validated by recording 2D ^1^H{^15^N} SOFAST-HMQC NMR spectrum.

Refolded KH23 was cleaved with AcTEV protease (Invitrogen, cat. no. 12575) following the manufacturer protocol to de-protect the *N*-terminal cysteine incorporated in KH2. Time points of the TEV digestion were analysed using the same HPLC conditions as the first step ligation reaction analysis. Cleaved products were analysed via microTOFQ electrospray mass spectrometer (Bruker Daltonics) Figs 2c and 3b.

Once digestion was complete the sample was purified on a pre-equilibrated C18 semipreparative reverse phase column using a 30–60% ACN gradient over 30 min. Again the purified cleaved ligated product was freeze dried and stored at −80 °C.

### KH1 and KH23 ligation

The KH1-KH23 ligation was performed using the same protocol as the previous ligation, except for the use of a Vydac 219TP DiPhenyl column (Grace) instead of a C18 column during the HPLC analysis. This was necessary to resolve the KH23 and KH123 construct species using a 25–35% ACN gradient over 30 min and a 1 ml/min flow rate.

After 4 h the sample underwent hydroxylamine treatment as for the KH2 KH3 ligation. The ligated KH123 construct was refolded following the same three-step dialysis as for the KH23 domains then a 2D ^1^H{^15^N} SOFAST-HMQC spectrum was recorded to validate protein folding.

### Mass Spectrometry

Purified proteins were characterized by MALDI-TOF MS on a Bruker Microflex using CHCA matrix (10 mg.ml-1 in ACN/H_2_O/TFA, 50:50:0.1) using the ion positive linear mode.

Samples analysed via electrospray mass spectrometry requiring desalting were purified using a C18 ZIPTIP (Millipore, cat. no. ZTC 185096) following standard protocol. The desalted protein was then infused into a microTOFQ electrospray mass spectrometer (Bruker Daltonics) at 3 µl/min using an electrospray voltage of 4.5 kV. Mass spectra were then de-convoluted using maximum entropy software (Bruker Daltonics).

### NMR spectroscopy

NMR data were recorded at 25 °C on a Bruker Avance III NMR spectrometer operating at 700 MHz ^1^H frequency and equipped with a 5 mm TCI cryoprobe (for ^1^H{^15^N} SOFAST-HMQC experiments) or a 600 MHz (for^15^N relaxation experiments). Samples were dialysed into NMR buffer (10 mM sodium phosphate (pH 6.4), 50 mM NaCl, 0.5 mM TCEP) with 10% D_2_O added to acquire the lock. 2D ^1^H{^15^N} SOFAST-HMQC experiments were recorded with 40 scans and 128 increments, processed using the NMRpipe suite of programs^35^ and analysed using the Sparky^36^ program. T1 measurements were obtained through preforming standard relaxation experiments^37^ with a time delay series of 10, 50, 100, 300, 600, 900 and 1200 ms. Data was analysed by using NMRPipe routines^35^ and time delay decay curves fitted using Gaussian models, also generating fitting error values as stated in supplementary data.

Data and materials generated during this study are available from the corresponding authors on reasonable request.

## Electronic supplementary material

Supplementary Information
