# Supplementary material for: A method for the unbiased and efficient segmental labelling of RNA-binding proteins for structure and biophysics
Source: Sci Rep. 2017 Oct 26;7:14083. doi: 10.1038/s41598-017-13950-8 (PMC5658380; doi:10.1038/s41598-017-13950-8)
Supplement: Supplementary file 1 — Supplementary Information [file 41598_2017_13950_MOESM1_ESM.pdf]

Supplementary Information

A method for the unbiased and efficient segmental labelling of RNA-binding proteins for structure and biophysics.

Christopher Gallagher, Fabienne Burlina, John Offer and Andres Ramos

Supplementary Table 1

Residue	T_1 / ms	Error / ms	Residue	T_1 / ms	Error / ms
G323	775	12	Q360	--	--
G324	693	9	D361	--	--
I325	748	10	D362	--	--
D326	773	11	G363	670	8
V327	737	10	T364	742	10
P328	--	--	G365	--	--
V329	848	13	P366	--	--
P330	--	--	E367	713	9
R331	651	8	K368	814	12
H332	--	--	I369	849	14
S333	782	11	A370	743	10
V334	672	8	H371	--	--
G335	531	6	I372	790	11
V336	719	9	M373	795	12
V337	746	10	G374	701	9
I338	740	10	P375	--	--
G339	718	9	P376	--	--
R340	--	--	D377	722	10
S341	--	--	R378	781	11
G342	592	9	C379	--	--
E343	--	--	E380	744	10
M344	--	--	H381	742	10
I345	738	10	A382	816	12
K346	738	11	A383	835	12
K347	--	--	R384	782	11
I348	740	10	I385	664	8
Q349	694	9	I386	514	6
N350	784	11	N387	775	11
D351	637	8	D388	787	11
A352	--	--	L389	805	12
G353	784	11	L390	776	11
V354	797	11	Q391	--	--
R355	761	11	S392	756	10
I356	777	11	L393	--	--
Q357	689	9	R394	524	7
F358	--	--	S395	--	--
K359	780	11	G396	759	11

Supplementary Figure 1

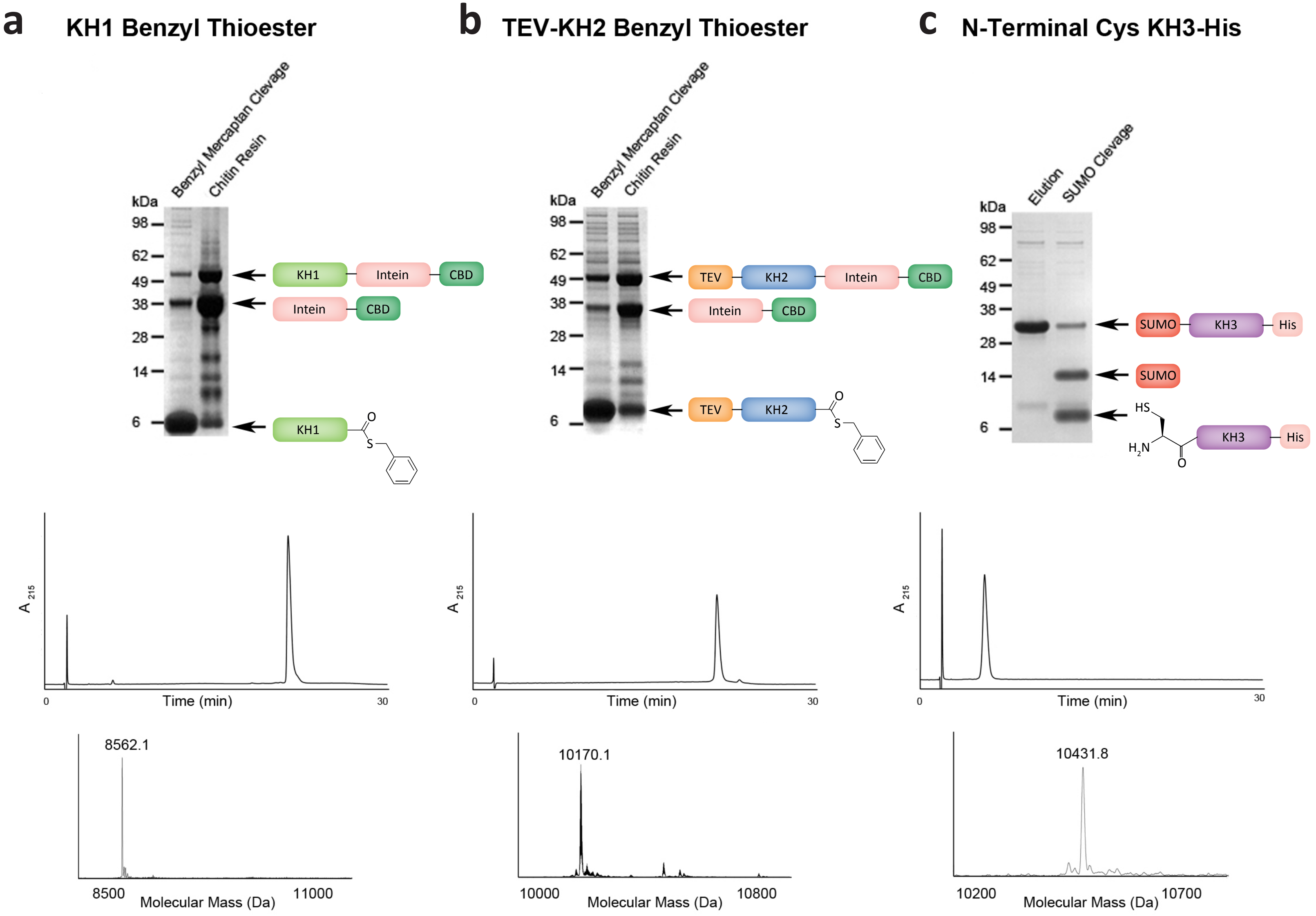

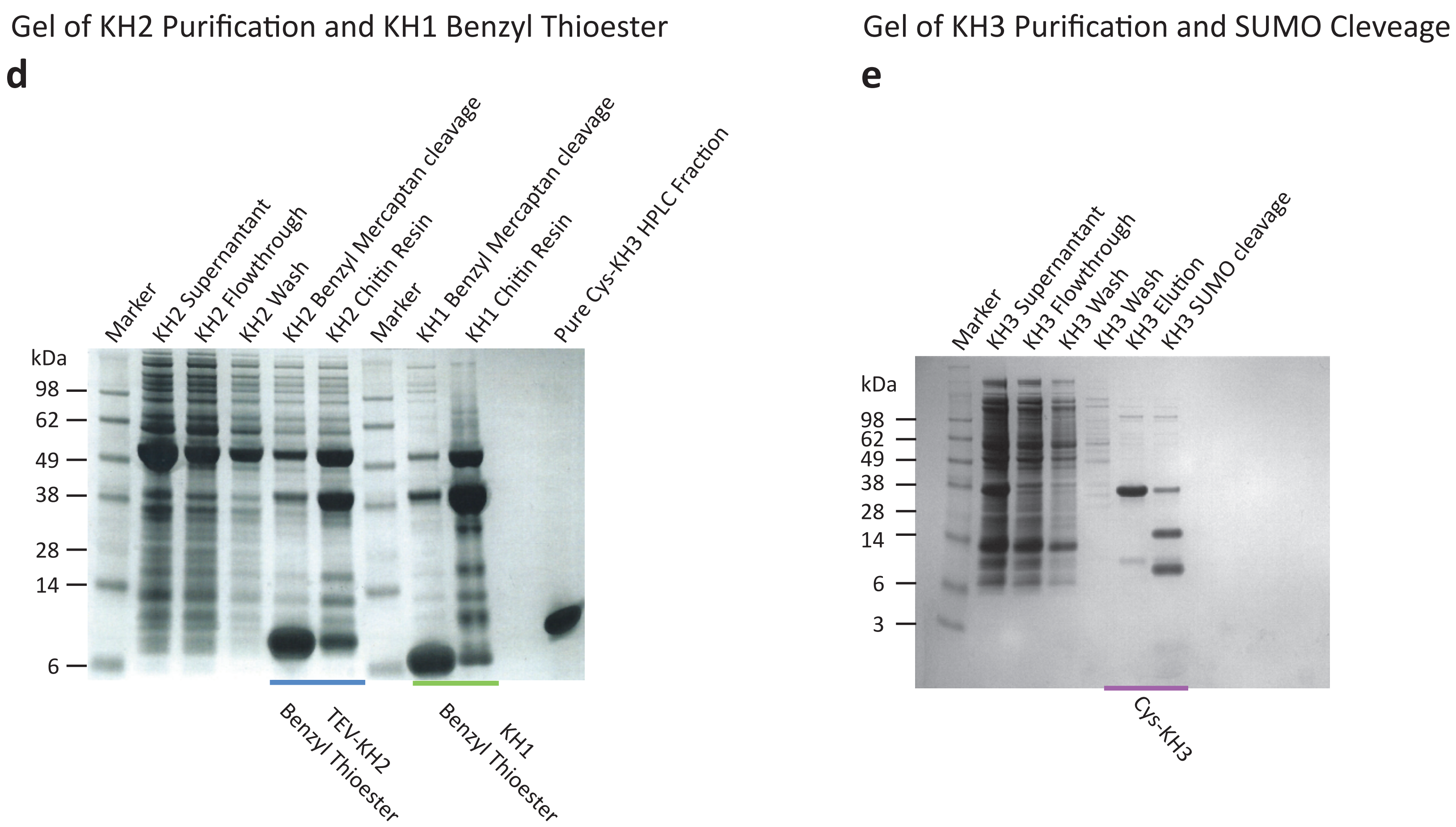

Figure S1 **Page one**. Unmasking of the reactive groups of the three KH domains. a) KH1 Benzyl thioester b) KH2 Benzyl thioester c) N-Terminal Cys KH3-His. For all three panels, Top: SDS-PAGE analysis of the reaction when close to completion. Middle: Analytical HPLC (C18 column) of the three KH domains used as reagents in the ligations. Bottom: Reconstituted electrospray mass spectrum showing the de-convoluted molecular mass of the purified reactive species. **Page two**. Complete original gel images: d) The full gel from which Figure S1a and S1b were created e) Cys-KH3 purification gel from which Figure S1c was taken.
